# Orthodontists’ Knowledge and Perception of the Prolonged Use of Rapid Palatal Expanders (RPEs) in the Saudi Arabian Population: A Cross-Sectional Study

**DOI:** 10.7759/cureus.71207

**Published:** 2024-10-10

**Authors:** Nancy Ajwa

**Affiliations:** 1 Department of Preventive Dentistry, College of Medicine and Dentistry, Riyadh Elm University (REU), Riyadh, SAU

**Keywords:** covid-19, expansion complications, orthodontics, palatal expander, tele-dentistry

## Abstract

Background

Orthodontic expansion using a rapid palatal expander (RPE), initiated early in life, is one approach to treating malocclusions. However, prolonged RPE use leads to negative consequences. The study aims to determine the perception and experience of orthodontists related to the prolonged use of RPE and their management.

Methods

A cross-sectional study was conducted among orthodontists (n = 1,125) in Saudi Arabia using an online survey. The questionnaire consisted of two sections. Section A included personal and demographic details, while Section B contained questions regarding participants' perception, knowledge, and experience related to the prolonged use of RPE and their management protocols among children aged 6 to 14 years. To assess differences in participants' perceptions and work experiences, including their approach toward tele-dentistry, the Chi-Square test was employed.

Results

The most prevalent reason for patients' prolonged usage of RPE was missing appointments due to multiple reasons, such as negligence, traveling (13.6%), or COVID-19 (9.6%). Among the types, the banded RPE was the most used (68.8%). The most common complications included buccal tipping of posterior teeth (72%), over-expansion (39.2%), and extrusion (25.6%).

Conclusions

Most orthodontists used a banded type of RPE, and the most common complication was buccal tipping of posterior teeth. There were no significant differences in complications when banded or bonded RPEs were used. The use of tele-dentistry and additional appliances to treat complications was reported by half of the participating orthodontists.

## Introduction

Maxillary constriction is caused by a lack of maxillary bone development in the transverse direction. A unilateral or bilateral posterior cross-bite is the most prominent clinical feature of maxillary constriction [1], which requires correction with orthodontic treatment in 10% to 15% of adolescents, irrespective of their gender and ethnicity [2]. This treatment has demonstrated superior effectiveness, and it is highly recommended to commence it during the child's natural growth phase. The most suitable orthodontic appliance for this type of treatment is a palatal expander [3-5].

The rapid palatal expander (RPE) is the most commonly used appliance for treating dental malocclusion due to its safety, reliability, and efficacy [6,7]. Primarily designed for orthopedic purposes, these expanders also induce some dental expansion, causing the teeth to tilt toward the labial side. As individuals age, this effect becomes more pronounced, accompanied by a thickening of the interdigitating palatine suture, which makes it progressively challenging to prioritize orthopedic benefits over dental benefits [8].

According to Venkateshwara et al., an RPE is a rigid device specifically designed for maximum dental anchoring. Its primary function is to expand the palate for 10 to 14 days, employing a jackscrew mechanism. This approach aims to optimize the orthopedic effects of the appliance. Notably, research indicates that this technique generates forces ranging from 3 to 10 pounds [9]. As a result, it must be capable of applying such forces without deforming to reduce the inclination of the teeth; when a patient's palate is unusually deep, the rigidity of the expander must be increased. Even after reaching adolescence, RPE screw activation decreases from 50% to almost a third of the quantity of transverse skeletal dimensions [10,11].

RPE has been reported to cause undesirable temporary or permanent side effects, such as buccal tipping of posterior teeth, extrusion, recession of periodontal tissue, relapse of expansion, fenestration of the buccal cortex, necrosis of palatal tissue, and failure in the opening of the suture. In addition, over the last two years, the population has undergone unusual conditions that have affected all aspects of life, especially during the COVID-19 pandemic. During the pandemic, dentists were required to limit their treatment to patients to avoid the possible spread of infection, given the high risk of infection in healthcare environments [12]. However, difficult communication has negative consequences, especially in orthodontics, due to uncontrolled patient performance and appliance activation rates [13].

This study aims to determine the perceptions among orthodontists regarding the complications of prolonged usage of RPE, in addition to comparing the management based on the practitioners' qualifications, clinical experiences, and work sectors.

## Materials and methods

Study design

A cross-sectional study was conducted among the orthodontists in Saudi Arabia using an online validated survey. The study obtained Institutional Review Board (IRB) approval (blinded for peer review). The approval number assigned to this study is "FRP/2022/496/890."

Sample size estimation

The sample size estimation was performed using the G*Power freeware (Heinrich-Heine-Universität Düsseldorf, Düsseldorf, Germany). Based on an effect size of 0.196, an alpha error probability of 0.05, a power of 0.95, and an allocation ratio of 0.88, a sample size of 1,125 was estimated. The effect size was determined by considering the mean difference of the Hiroshima University Dental Behavioral Inventory (HU-DBI) scores [14], which ranged from 3 to 9, as reported in a previous study [15].

Study instrument

A pre-validated questionnaire was used, with modifications as required for the current study (Appendix A) [16]. The face validity of the modified questionnaire was determined by subject matter experts who are experienced researchers at Riyadh Elm University. The questionnaires comprised personal and professional questions, along with demographic details. In addition, another 11 questions were related to the participants' experiences, perceptions, and knowledge about the consequences of prolonged use of RPE, the most common complications, and their management techniques. The participating orthodontists, consultants, specialists, and residents completed the questionnaires. A pilot study was conducted before starting the main study to assess intra-examiner reliability using a subset of the study population; a value greater than 0.80 from the kappa test showed an acceptable range. Informed consent was obtained from the participating orthodontists, and the survey forms were distributed personally to the orthodontists during their clinical hours, where feasible, or via official emails and social media platforms.

Statistical analysis

Data analysis was conducted using IBM SPSS Statistics for Windows, Version 22 (Released 2013; IBM Corp., Armonk, NY, USA). Descriptive statistics were utilized to present demographic details, providing a comprehensive overview of the study participants. To assess differences in participants' perceptions and work experiences, including their approach toward tele-dentistry, the Chi-Square test was employed. A p-value of less than 0.05 was considered statistically significant.

## Results

**Figure 1:**
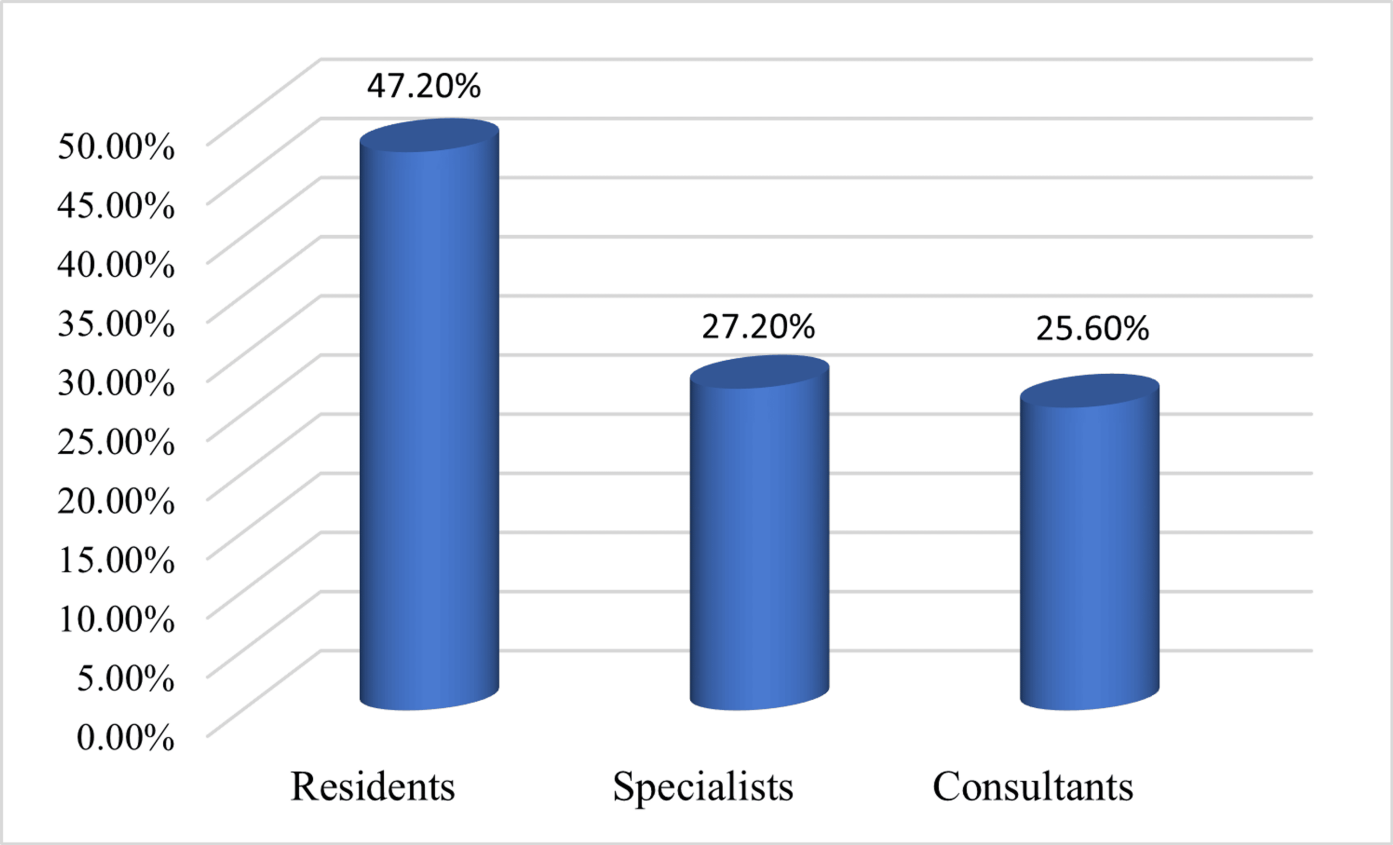
Distribution of orthodontists (percentages) according to work experience

A total of 1,125 orthodontists participated in this survey-based study, of which 47.20% were residents, 27.20% were specialists, and 25.60% were consultants. Regarding their working sector, 28% belonged to the private sector, whereas 72% were government employees. Regarding clinical experience, 58.4% had experience of less than five years, 24% had 5 to 10 years, and 17.6% had more than 10 years, as shown in Figure 1.

The overall responses, along with their frequencies, to the questionnaire are shown in Table 1. Around 47.2% of the participants reported using RPE in routine practice once every few months, and 51.2% stated that they had experienced patients using RPE for a prolonged period (more than six months). The most common reason behind missing appointments was reasons other than COVID-19 (9.6%) and traveling (13.6%). Regarding the patients' gender and age groups treated by the participating orthodontists, most were females aged 6-14 years. When inquired about the type of RPE, 68.8% had banded RPE, whereas 31.2% had bonded RPE. Concerning the number of cases with complications related to RPE, the majority (36.8%) stated one patient. The most common complication from prolonged RPE use was buccal tipping of posterior teeth, seen among 72% of the patients, followed by over-expansion (39.2%) and relapse of expansion (31.2%). About 49.6% of orthodontists reported having contacted their patients using tele-dentistry, 58.4% reported removing the appliance immediately, 48.8% indicated they used another appliance to address the complications, and 36.8% used elastics to resolve the issues.

**Table 1:** Overall responses to the survey questions RPE: Rapid palatal expander

Category	Count	Percentage
Frequency of RPE use in routine practice		
Daily	81	7.20%
Weekly	207	18.40%
Monthly	306	27.20%
Once every few months	531	47.20%
Have you experienced patients using RPE for a prolonged period of time?		
Yes	576	51.20%
No	549	48.80%
If yes what was the reason?		
No reasons	549	48.80%
Missed appointment due to COVID-19	108	9.60%
Missed appointment due to other reason	315	28.00%
Missed appointment due to traveling	153	13.60%
Gender of the patients treated		
Male	558	49.60%
Female	567	50.40%
Age of the patients treated		
6-8 years	171	15.20%
9-11 years	468	41.60%
12-14 years	486	43.20%
Which type of RPE did you use?		
Banded	774	68.80%
Bonded	351	31.20%
How many cases with complications have you faced?		
None	270	24.00%
One	414	36.80%
Two	261	23.20%
More than two	180	16.00%
What is the most common complication from prolonged use of RPE?		
Buccal tipping of posterior teeth	461	41.00%
Over expansion	236	21.00%
Relapse of expansion	214	19.00%
Extrusion	169	15.00%
Failure of opening the suture	23	2.00%
Other	22	2.00%
Did you contact your patients using tele-dentistry during the time of their absence?		
Yes	558	49.60%
No	567	50.40%
Did you remove the appliance immediately for the patient?		
Yes	657	58.40%
No	468	41.60%
Did you use another appliance to overcome the complications?		
Yes	549	48.80%
No	576	51.20%
Did you use elastics to overcome the complications?		
Yes	414	36.80%
No	711	63.20%

The Chi-Square test of independence was conducted to compare the survey responses among orthodontists with different qualifications: residents, specialists, and consultants. The analysis revealed no statistically significant differences in the frequency of RPE use in routine practice across the three groups (p = 0.53). Similarly, the responses regarding experiencing patients using RPE for a prolonged period, the reasons for such prolonged use, and the gender of the patients treated showed no significant variation among the different qualifications (p = 0.427). Furthermore, there were no significant differences in the type of RPE used, the number of cases with complications, and the most common complications reported (p = 0.429). Additionally, the use of tele-dentistry during patient absence, the immediate removal of the appliance, and the use of another appliance or elastics to overcome complications did not significantly differ across the qualifications of orthodontists (p = 0.101) (Table 2).

**Table 2:** Comparison of survey responses between residents, specialists, and consultants RPE: Rapid palatal expander

		Residents, n = 531	Specialists, n = 306	Consultants, n = 288	p-value
Frequency of RPE use in routine practice	Daily	47.0%	23.0%	30.0%	0.53
	Weekly	41.0%	19%	40.0%	
	Monthly	45%	20%	35%	
	Once every few months	30.0%	45.0%	25%	
Have you experienced patients using RPE for a prolonged period of time?	51.20%	62.2%	7.8%	30.0%	0.705
	48.80%	20.0%	37.5%	42.5%	
If yes what was the reason?	No reasons	66.7%	3.3%	30.0%	0.57
	Missed appointment due to COVID-19	48.6%	31.4%	20.0%	
	Missed appointment due to other reason	20.0%	47.1%	32.9%	
	Missed appointment due to traveling	30.0%	45.0%	25%	
Gender of the patients treated	Male	65.2%	4.8%	30.0%	0.427
	Female	20.0%	39.2%	40.8%	
Age of the patients treated	6-8 years	20%	45%	35%	0.567
	9-11 years	65%	23.0%	12.0%	
	12-14 years	21.0%	41.0%	38.%	
Which type of RPE did you use?	Banded	48.6%	31.4%	20.0%	0.898
	Bonded	20.0%	17.9%	62.1%	
How many cases with complications have you faced?	None	41.0%	19%	40.0%	0.429
	One	43.0%	37.0%	20.0%	
	Two	20.0%	48.6%	31.4%	
	More than two	21.0%	41.0%	38.%	
What is the most common complication from prolonged use of RPE?	Buccal tipping of posterior teeth	66.7%	3.3%	30.0%	0.181
	Over expansion	29.7%	70.3%	0.0%	
	Relapse of expansion	0.0%	65.4%	34.6%	
	Extrusion	43.0%	37.0%	20.0%	
	Failure of opening the suture	66.7%	3.3%	30.0%	
	Other	16.5%	43.0%	40.5%	
Did you contact your patients using tele-dentistry during the time of their absence?	Yes	65.2%	4.8%	30.0%	0.935
	No	30.0%	39.2%	30.8%	
Did you remove the appliance immediately for the patient?	Yes	60.8%	19.2%	20.0%	0.574
	No	30.0%	18.5%	51.5%	
Did you use another appliance to overcome the complications?	Yes	66.7%	3.3%	30.0%	0.616
	No	30.0%	20.0%	50.0%	
Did you use elastics to overcome the complications?	Yes	30.0%	45.0%	25%	0.101
	No	16.5%	43.0%	40.5%	

A comparison of survey responses between orthodontists working in the government sector and those in the private sector was conducted using the Chi-Square test. The results indicated no statistically significant differences in the frequency of RPE use in routine practice (p = 0.520). However, two significant differences were found: the age group of patients treated (p = 0.007) and the immediate removal of the appliance for the patient (p = 0.006), with private practitioners showing higher percentages. Additionally, significant differences were observed in the use of another appliance to overcome complications (p = 0.015) and in the use of elastics to address complications (p = 0.003), with private practitioners being more likely to adopt these measures (Table 3).

**Table 3:** Comparison of survey responses based on work sector (Government vs Private) *Statistically significant at 0.05 RPE: Rapid palatal expander

Survey questions	Responses	Government	Private	p-value
Frequency of RPE use in routine practice	Daily	15.7%	7.5%	0.520
	Weekly	65.7%	7.5%	
	Monthly	8.6%	29.4%	
	Once every few months	10.0%	55.6%	
Have you experienced patients using RPE for a prolonged period?	Yes	49.0%	32.2%	0.076
	No	51.0%	67.8%	
If yes, what was the reason?	No reasons	50.0%	28.9%	0.056
	Missed appointment due to COVID-19	15.0%	13.3%	
	Missed appointment due to other reason	20.0%	38.9%	
	Missed appointment due to traveling	15.0%	18.9%	
Gender	Male	48.0%	30.0%	0.478
	Female	52.0%	70.0%	
Age	6-8 years	8%	31%	0.007*
	9-11 years	41%	31%	
	12-14 years	41%	38%	
Did you remove the appliance immediately for the patient?	Yes	51%	77%	0.006*
	No	49%	23%	
Did you use another appliance to overcome the complications?	Yes	42%	66%	0.015*
	No	57.8%	34.3%	
Did you use elastic to overcome the complications?	Yes	28.8%	57.1%	0.003*
	No	71.2%	42.9%	

The survey responses were also compared based on the clinical experience of orthodontists (less than five years, 5-10 years, more than 10 years). The Chi-Square test revealed statistically significant associations between highly experienced practitioners and the complications encountered. Specifically, significant differences were found in the immediate removal of the appliance for patients (p = 0.001), with less experienced orthodontists being more likely to remove the appliance immediately. Additionally, significant differences were observed in the use of another appliance to overcome complications (p = 0.011) and in the use of elastics to address complications (p = 0.012), with more experienced orthodontists more frequently employing these measures (Table 4).

**Table 4:** Comparison of survey responses based on clinical experience *Statistically significant at 0.05

Survey questions	Response	Less than 5 years	5 to 10 years	More than 10 years	p-value
Did you remove the appliance immediately for the patient?	Yes	80.8%	19.2%	0.0%	0.001*
	No	0.0%	38.5%	61.5%	
Did you use another appliance to overcome the complications?	Yes	38.3%	56.6%	72.7%	0.011*
	No	61.7%	43.4%	27.3%	
Did you use elastic to overcome the complications?	Yes	28.7%	36.6%	63.6%	0.012*
	No	71.3%	63.4%	36.4%	

## Discussion

The current study aimed to determine the perceptions and knowledge of orthodontists regarding the complications associated with the prolonged use of RPE. The participants' methods of dealing with complications were assessed and observed. Moreover, comparisons were also made based on qualification, clinical experience, work sector, and the type of RPE.

Notably, the study identified significant correlations with highly experienced practitioners in two areas. First, experienced orthodontists were more inclined to use alternative appliances to address complications, indicating their openness to exploring diverse treatment options. Second, they showed a higher tendency to apply elastics in managing orthodontic issues effectively.

The research also explored factors related to work sectors, revealing significant differences between the two areas. First, the age group of patients influenced the type of orthodontic treatment provided, indicating different approaches based on patient age. Second, private practitioners showed significantly higher rates of immediate appliance removal compared to others, suggesting distinct practices in this aspect. Moreover, there is a significant correlation between the use of other appliances, including elastics.

Furthermore, the study compared orthodontists' responses based on their qualifications and found no statistically significant differences for any of the questions. This suggests that orthodontists' qualifications did not have a significant impact on their responses to the survey questions.

Additionally, it can be noted from the findings that one of the complications of prolonged use of RPE is an increase in the vertical dimension, which was found in around 15% of the patients. However, no statistically significant difference was achieved when comparing this based on the bonded vs. banded type of RPEs. A similar study conducted by Reed et al. revealed similar findings, in which they found that the banded RPE appliance caused only 1 mm of additional vertical changes compared to the bonded group [17]. Furthermore, similar results were obtained by Martins et al., who compared the root resorption ability between the banded and bonded RPEs [18]. On the contrary, a study by Olmez et al. revealed that the tipping of first molar and premolar teeth was significantly higher in the banded group than in the bonded group [19]. These findings conflicted with the current result.

It has been proven that RPE is one of the techniques most broadly used in orthodontics. Since the palate relates to the nasal base, nasal concerns have been noted due to this process, such as lowering the palatine vault, lengthening the nasal septum, and lateralizing the inferior nasal turbinate. These nasal alterations due to RPE may improve the respiratory pattern [19-21]. The current results show that nose-related complications due to the prolonged use of RPE were only in the 2-4% range. This number is negligible compared to the other commonly occurring complications [22-24].

In the literature, Kiliç and Oktay [25] and Putrino et al. [26] have examined the relationship between the use of RPE and nasal complications. Their studies have revealed that, even though orthodontic treatment aims to correct dental and skeletal discrepancies, the treatment consequences of RPE could also impact naso-respiratory complications among growing children. However, it is important to note that this aspect of RPE has been investigated in a limited number of studies with relatively low levels of data. Gurel et al. conducted a study to assess the long-term variations in maxillary arch widths, overjet, and overbite in patients who were treated with rapid maxillary expansion (RME). It was revealed that RME resulted in an outright increase in maxillary arch widths, overjet, and overbite in patients treated with RME. Their findings revealed that RME led to a significant increase in maxillary arch widths. However, some of the width increases caused by RME were overturned during fixed appliance treatment, and a significant extent of relapse arose in the long term, the greatest being in inter-canine width. Similar findings were observed in our study, where more than 31.2% of patients presented with a relapse of expansion [27].

During the quarantine and pre-vaccine period of COVID-19, dental professionals were on the verge of being exposed to infection, and the majority of dental clinics specializing in routine dental treatment were closed; only emergency treatment was delivered to patients [28]. This gap in attending appointments on time was tackled by technological developments, which significantly influenced medicine. Due to the increased usage of smartphones and associated software applications, clinical information exchange was aided between patients and orthodontists, known as "tele-dentistry" [29]. Our results revealed that almost half of the orthodontists utilized tele-dentistry as a tool for communication during the time of COVID-19. Berndt et al. exhibited a significantly improved outcome among patients receiving orthodontic consultations using tele-dentistry [30]. A research study conducted in Saudi Arabia emphasized the significance of factors associated with the patient-doctor relationship and environmental conditions in determining satisfaction levels with RME therapy. The study revealed that, in general, parents exhibited greater satisfaction with pediatric dentists compared to orthodontists in terms of the treatment results offered to their children [6].

The study paved the way for understanding the perspectives of orthodontists practicing in Saudi Arabia. It effectively identified significant correlations between orthodontists' experience and their preferred methods for managing RPE complications, providing valuable insights into diverse treatment approaches. However, one of the study's limitations was the absence of questions regarding prolonged treatment with RMEs. Additionally, like any other questionnaire-based study, there is a risk of social desirability bias in the question related to tele-dentistry, where dentists may wish to convey that they have engaged with the patient, although this is not measured using any form of data. Another limitation of the study is the use of closed-ended questions concerning management techniques. If these questions had been open-ended, valuable information could have been gathered regarding the alternative treatment procedures adopted by different orthodontists working in various sectors, based on resource availability and patient cooperation.

Future qualitative research in this area is recommended to overcome these limitations and gain a deeper understanding. Such research would provide more comprehensive data and additional insights into the existing literature, enabling an analysis of the effectiveness of management techniques based on orthodontists' perceptions.

## Conclusions

Several key findings emerged from the current study. Firstly, the most prevalent complication associated with RPE was the buccal tipping of posterior teeth, with most orthodontists preferring the banded type of RPE. Secondly, the perceptions of RPE among orthodontists were consistent regardless of their qualifications, clinical experience, or work sector, indicating a shared understanding and approach to RPE complications. Thirdly, no significant differences in complications were found between the banded and bonded types of RPE. Lastly, approximately 50% of the orthodontists reported utilizing tele-dentistry and additional appliances to manage complications, demonstrating adaptability and resourcefulness in their practice. These findings contribute valuable insights into the practices and perceptions of orthodontists regarding RPE use and management.

## Appendices

Appendix A

Survey questions

1) Frequency of RPE use in routine practice

Daily

Weekly

Monthly

Once every few months

2) Have you experienced patients using RPE for a prolonged period of time?

Yes

No

3) If yes, what was the reason?

No reasons

Missed appointment due to COVID-19

Missed appointment due to other reason

Missed appointment due to traveling

4) Gender of patient

Male

Female

5) Age group of patient

6-8 years

9-11 years

12-14 years

6) Which type of RPE did you use?

Banded

Bonded

7) How many cases (with complications) have you faced?

None

One

Two

More than two

8) Most common complication from prolonged used of RPE?

Buccal tipping of posterior teeth

Over expansion

Relapse of expansion

Extrusion

Failure to open the suture

Other

9) Did you contact your patients using tele-dentistry during the time of their absence?

Yes

No

10) Did you remove the appliance immediately for the patient?

Yes

No

11) Did you use another appliance to overcome the complications?

Yes

No

12) Did you use elastic to overcome the complications?

Yes

No
